# Working toward a Multi-Program Strategy in Fall Prevention

**DOI:** 10.3389/fpubh.2014.00254

**Published:** 2015-04-27

**Authors:** Bonita Lynn Beattie

**Affiliations:** ^1^National Council on Aging, Washington, DC, USA

**Keywords:** fall prevention, enlisting community programs that work, community health services, healthy aging, falls free initiative

The exceptional compilation of healthy aging articles contained within this Research Topic are timely, and highlight many important ongoing health care reform initiatives to improve the healthy behaviors of older adults and aging boomers. The national discussion understandably focuses on chronic conditions including cost containment, improved patient outcomes, and quality of life measures. However, I would suggest that the prevention of older adult falls and related injuries should be an integral part of the discussion. By broadening the discussion of effective management of chronic diseases and focusing on how to help inform, educate, and support aging Americans, we could also reduce the growing number of falls and falls-related injuries and deaths in this vulnerable population (1).

While the evidence is strong that a small number of targeted prevention programs have significantly reduced falls in older adults, few of these programs followed participants for longer than 12 months (2). However, in the absence of long-term outcome data tracking the maintenance of behavior changes, it is difficult to evaluate if we are promoting long-term healthy behaviors or just forestalling the onset of a fall.

The evidence is equally strong for linking the growing number of chronic conditions in older adults to falls. Chronic disease can significantly increase the risk of a variety of factors associated with those diseases. This includes, but is not limited to functional limitations and disabilities; chronic pain; sensory deprivations; vision effects; and balance and gait disturbances. Chronic disease manifestations may also increase the risk of falls through indirect effects such as reduced physical activity level, reduced social activities, and potential depression or anxiety. Medications to treat chronic diseases can also lead to an increased risk of falls through both the absolute number taken and the potential interactions (3–6).

Research strongly suggests that people who exercise regularly live longer and healthier lives. Being physically active and following an exercise program can reduce the risk of developing some diseases and disabilities that often occur with age. Strength exercises build muscles and reduce the risk of osteoporosis. Flexibility or stretching exercises help keep the body and joints flexible and often help to modulate pain (7). Not surprisingly, exercise – especially strength, balance, and flexibility – is a key strategy in reducing the risk of falls and serious injury.

Seminal research by Tinetti and colleagues noted that the cumulative number of falls risks (including but not limited to declining strength; balance/gait issues; vision changes; postural blood pressure; depression; arthritis; foot problems; multiple medications; and environmental hazards) mattered (8). So, it seems that the questions worth exploring are:

- Can we make a strong case for the fall prevention contributions of community programs effective in helping older adults make behavior changes to enhance the management of their chronic conditions?
- Can we consider a multi-program, longer-term community strategy that helps to maintain behavior change, promotes physical activity, and helps to better manage medications and chronic conditions as a longer-term fall prevention strategy?
- How will seniors/caregivers view this change in strategy? More importantly how can we recruit growing numbers of senior participants, program leaders, and mentors?
- How can we capture outcomes to promote the reimbursement of programs that can reduce health care costs and promote quality of life?

## Making a Sustainable Difference

It is evident that adequately managing expressions of chronic conditions and supportive medication regimens can affect the risk of falls and fall-related injuries in older adults. I believe that there is urgency to broaden the discussions on chronic disease management and how to best apply disease management guidelines to fall prevention. Further, there is an opportunity to capitalize on the investments of the U.S. Administration on Community Living in the dissemination of sustainable, evidence-based health promotion, and chronic disease self-management programs.

As the population of elderly grows to over 70 million by 2030 (9), there is value, even an urgency, to enlist community evidence-based programs and services to offer older adults the opportunity to better manage their chronic disease, enhance their level of physical activity, and modify their risk of falls and injury.

What is needed now is a more inclusive approach to the effective management of chronic disease and reduction of fall risk; an approach that values and enfolds the broad spectrum of healthy aging program offerings. I believe that by providing evidence-based prevention programs to help older adults and their caregivers make better choices, improve their health, and increase their quality of life will ultimately affect the rate of elderly falls.

## Conflict of Interest Statement

The author declares that the research was conducted in the absence of any commercial or financial relationships that could be construed as a potential conflict of interest.

This paper is included in the Research Topic, “Evidence-Based Programming for Older Adults.” This Research Topic received partial funding from multiple government and private organizations/agencies; however, the views, findings, and conclusions in these articles are those of the authors and do not necessarily represent the official position of these organizations/agencies. All papers published in the Research Topic received peer review from members of the Frontiers in Public Health (Public Health Education and Promotion section) panel of Review Editors. Because this Research Topic represents work closely associated with a nationwide evidence-based movement in the US, many of the authors and/or Review Editors may have worked together previously in some fashion. Review Editors were purposively selected based on their expertise with evaluation and/or evidence-based programming for older adults. Review Editors were independent of named authors on any given article published in this volume.
